# The Impact of COVID-19 on Bicycle-Related Injuries Managed at a Level 1 Major Trauma Center in London, United Kingdom

**DOI:** 10.7759/cureus.59465

**Published:** 2024-05-01

**Authors:** Isabella Drummond, Duncan Coffey, Sarah Bolton, Kyra Edwards, Abdallah Habiba, Ahmed M ElBaz, Omar Haggag

**Affiliations:** 1 Trauma and Orthopaedics, Barts Health NHS Trust, London, GBR

**Keywords:** major trauma center, push bike, general trauma surgery, covid-19, cycling

## Abstract

Introduction

The COVID-19 pandemic changed peoples' travel behaviors; an uptake in cycling was observed in the United Kingdom. The aim of this study was to assess the cycling-related orthopedic injuries presented to a major trauma center (MTC) before and during the COVID-19 pandemic.

Method

This retrospective observational single-center study analyzed referrals to the orthopedic department during a matched two-month period in 2019 and 2020. Data were collated on cycling-related injuries including demographic variables, mechanism of injury, anatomical area of injury, and the management of injury. The data were compared and statistical analysis was performed using the Pearson Chi-squared test to assess for significance.

Results

A total of 2409 patients were referred to the orthopedic department with injuries. A 35.6% decrease in total referrals was made during the COVID-19 pandemic. Analysis of cycling-specific injuries demonstrated a statistically significant increase in referrals to the orthopedic department during the COVID-19 pandemic. A statistically significant difference in upper limb trauma was also observed during the COVID-19 pandemic. Patterns of management, namely operative vs. non-operative management, did not demonstrate a difference in the two time periods.

Discussion

This study highlights that during the COVID-19 pandemic, cycling behavior changed with more patients suffering orthopedic injuries as a result. Orthopedic departments may need to plan for this change in behaviors with more capacity being created to manage the demand.

Conclusion

Cycling-related injuries referred to the orthopedic department increased during the pandemic.

## Introduction

The first patient with the novel coronavirus SARS-COV-2 was admitted to hospital in December 2019 in Wuhan, China [1]. After this, infection spread rapidly and the World Health Organisation declared a pandemic on 11th March 2019 [2]. The UK government responded by initiating a nationwide lockdown on 23rd March 2020 [3]. The lockdown aimed to reduce viral transmission, ensuring that the NHS was able to continue to provide healthcare without being overrun.

During lockdown, many people turned to cycling to avoid public transport. Cycling was also a way of keeping fit, with many exercise facilities such as gyms and outdoor recreation closing. The increase in people cycling was acknowledged by the UK Government, which invested £2 billion in May 2020 into walking and cycling; they also facilitated emergency cycle lanes and introduced the "fix your bike scheme" allowing people to apply for a £50 voucher for bicycle repairs [4]. Bicycle shops saw new bicycle sales soar and the so-called "bike boom" ensued [5].

Bicycle-related injuries are a cause of morbidity and mortality across the world. Cycling injuries, whether on the road or a cycle path, can be serious and life-changing; traumatic injuries including pelvic trauma, spinal trauma, and head injuries are well documented [6,7].

An Australian analysis of cycling injuries in a major trauma center (MTC) demonstrated that 78% of injured cyclists who were classified as major trauma had returned to work after six months [6]. However, only a third of participants reported a complete functional recovery, highlighting a significant economic burden following a serious cycling-related collision.

An epidemiological study from The Netherlands reviewed 1986 patients with bicycle-related injuries over a 10-year period and found a mortality rate of 5.7% in those admitted to hospital [7]. In addition, they showed that 41% of these patients had multiple injuries and highlighted the fact that cyclists admitted to the hospital had a high mortality rate.

Despite the risks associated with cycling, Hartog et al. estimated that the beneficial effect of cycling due to increased physical activity resulted in nine times more gain in life years than the loss from inhaled air pollution and traffic collisions [8].

The aim of this study was to assess whether the COVID-19 pandemic changed the pattern and management of bicycle-related orthopedic injuries in a level 1 MTC in London.

## Materials and methods

Patient data 

For this retrospective study, we reviewed all patients referred to the orthopedic department from a single Level 1 MTC in London from May 2019 to June 2019. This was compared to a similar cohort taken from May 2020 to June 2020. This corresponded to a period before the pandemic and a matched period during the pandemic when the UK lockdown was established.

All patients referred to the orthopedic department were included in the data. All adults (over 16 years of age) referred directly to the orthopedic department or indirectly through the virtual fracture clinic were included. Data was collected from electronic take lists and virtual fracture clinic lists. 

Outcome measures

The total number of patients in the study was 2409. From these patients, the notes were analyzed to see if the mechanism of injury was related to a bicycle. Pediatric patients, injuries from motorized E-bikes, and patients without orthopedic injuries were excluded.

The data was collected in Excel and included the date of birth, the date of injury, and sex. The age was calculated at the date of injury. The mechanism was split into fall from bike, cyclist vs. object, cyclist vs. car, and cyclist vs. large vehicle (including buses, lorries, and vans). Each patient’s notes were assessed for the fracture pattern and put into the general categories of upper limb and lower limb injuries initially. This was further categorized into specific categories. The management was divided into conservative and operative management, which included K-wires, open reduction and internal fixation, external fixators, joint replacements, and washout and debridement.

Statistical analysis 

All statistical analysis was performed using R statistic software. Continuous variables were described using means and ranges. Categorical variables were described using counts and percentages. Categorical outcomes were compared using Pearson’s chi-squared test. The significance threshold was set at P<0.05. 

Ethics approval 

Ethical approval was not sought for this study, as it was a retrospective observational study and the data was collected in line with the GMC guidelines for good clinical practice.

## Results

The total number of patients included in the study was 2409; this represents the total number of patients referred for all injuries to the orthopedic department over both periods. In 2019 the number of patients referred to the orthopedic service was 1751 compared with 1128 patients referred in 2020, representing a 35.6% decrease in the total number of referrals during lockdown (see Table 1). 

**Table 1:** Summary of results.

	May 2019	June 2019	May 2020	June 2020
Upper limb injuries	34 (77%)	43 (72%)	40 (77%)	53 (80%)
ACJ disruption	3 (7%)	5 (8%)	4 (8%)	2 (3%)
Both forearm fracture	0 (0%)	1 (2%)	0 (0%)	3 (5%)
Carpal bone fracture	4 (9%)	5 (8%)	1 (2%)	7 (11%)
Clavicle fracture	3 (7%)	6 (10%)	5 (10%)	3 (5%)
Distal radius fracture	0 (0%)	8 (13%)	6 (12%)	10 (15%)
Elbow dislocation	0 (0%)	0 (0%)	0 (0%)	1 (2%)
Humerus fracture	1 (2%)	1 (2%)	3 (6%)	3 (5%)
Olecranon fracture	0 (0%)	2 (3%)	2 (4%)	1 (2%)
Radial head fracture	10 (23%)	6 (10%)	10 (20%)	14 (21%)
Scapular fracture	1 (2%)	1 (2%)	1 (2%)	0 (0%)
Shoulder dislocation	2 (5%)	2 (3%)	0 (0%)	0 (0%)
Shoulder fracture dislocation	0 (0%)	0 (0%)	1 (2%)	0 (0%)
Supracondylar fracture	0 (0%)	0 (0%)	0 (0%)	1 (2%)
Ulnar fracture	0 (0%)	1 (2%)	0 (0%)	1 (2%)
Upper limb soft tissue injury	10 (23%)	5 (8%)	7 (13%)	7 (11%)
Lower limb injuries	10 (23%)	10 (17%)	9 (17%)	10 (15%)
Ankle fracture	2 (5%)	1 (2%)	2 (4%)	0 (0%)
Femur fracture	1 (2%)	0 (0%)	0 (0%)	0 (0%)
Lower limb soft tissue injuries	3 (7%)	2 (3%)	1 (2%)	1 (2%)
Neck of femur fracture	0 (0%)	1 (2%)	0 (0%)	1 (2%)
Patella fracture	0 (0%)	0 (0%)	0 (0%)	1 (2%)
Pelvic fracture	2 (5%)	3 (5%)	2 (4%)	1 (2%)
Periprosthetic fracture	1 (2%)	0 (0%)	0 (0%)	0 (0%)
Phalanx fracture	1 (2%)	0 (0%)	0 (0%)	0 (0%)
Tarsal fracture	0 (0%)	2 (3%)	0 (0%)	3 (5%)
Tib/fib fracture	0 (0%)	1 (2%)	0 (0%)	1 (2%)
Tibial plateau	0 (0%)	0 (0%)	4 (8%)	2 (3%)
Other injuries	0 (0%)	7 (12%)	3 (6%)	3 (5%)
Foreign bodies	0 (0%)	1 (2%)	0 (0%)	0 (0%)
Polytrauma	0 (0%)	5 (8%)	3 (6%)	3 (5%)
Spine fractures	0 (0%)	1 (2%)	0 (0%)	0 (0%)

**Figure 1:**
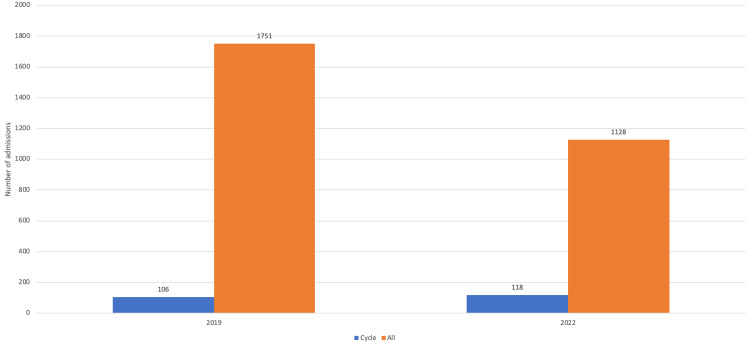
Analysis of the number of bicycle-related injuries to the proportion of the total number of referrals.

There was a statistically significant increase in bicycle-related trauma when compared to the proportion of total referrals in 2020 (10.5%) vs. 2019 (6.1%; p=0.000089) (Figure 1). 

The average age of patients in 2019 was 34.8 years vs. 31.5 years in 2020. Although there was a decrease in the average age, this was not found to be statistically significant (p=0.177). 

There was an increase in the number of women involved in cycling collisions with 24 females presenting in 2019 and 34 in 2020; however, this was not significant (p=0.349). 

**Figure 2:**
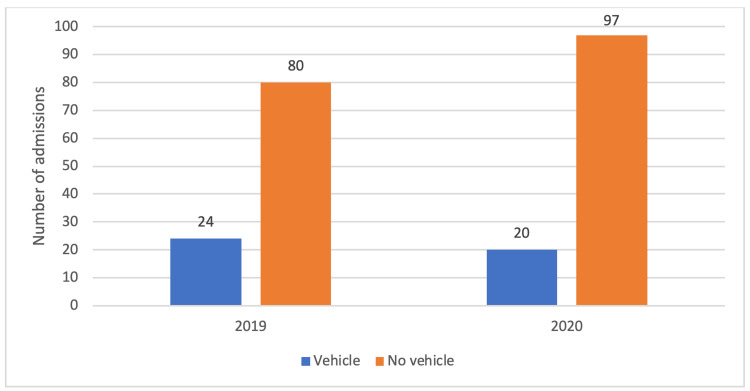
A graph comparing the mechanism of bicycle-related accidents in 2019 and 2020.

When analyzing the mechanism of injury, there was no statistical difference in the mechanism (cyclist vs. no vehicle and cyclist vs. vehicle) between the patients in 2019 and 2020 (Figure 2). There was a trend toward increased bicycle collisions that were not vehicle-related in 2020 but this was not statistically significant (p=0.346).

**Figure 3:**
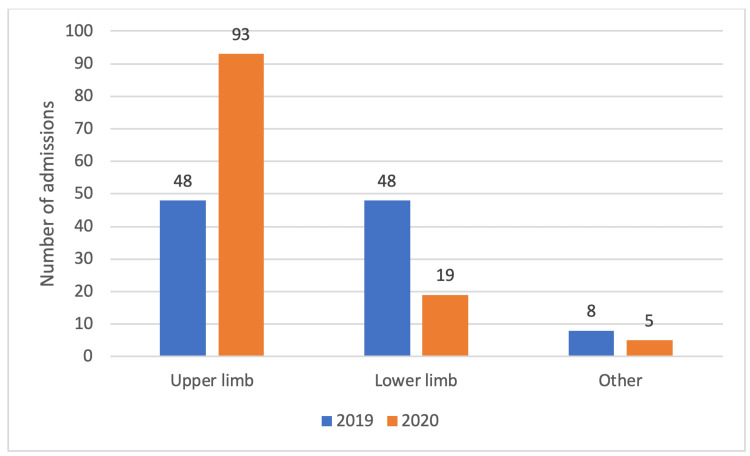
A graph demonstrating the pattern of injuries by anatomical location.

The anatomical location of the injury was assessed and grouped by location, namely upper limb, lower limb, and other injuries (Figure 3). The most frequently occurring injuries seen were radial head fractures with 15.1% (n=16) patients presenting in 2019 and 20.3% (n=24) in 2020, respectively. Analysis of injury type by anatomical location demonstrated a statistically significant increase (p=0.00000142) in the amount of upper limb trauma during 2020. 

**Figure 4:**
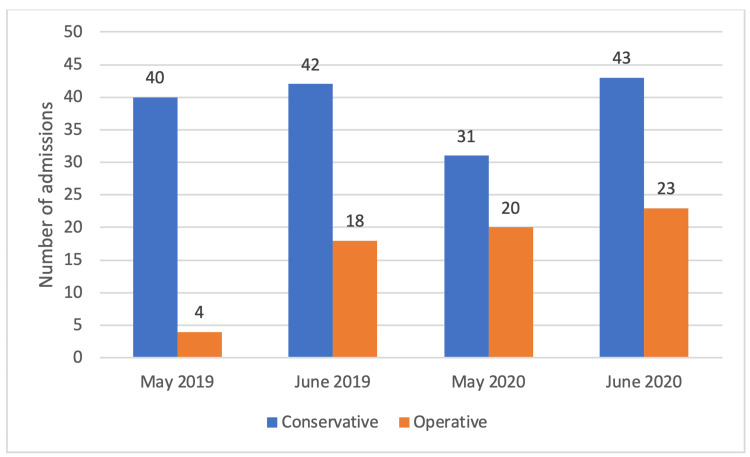
A graph comparing the management of cycling-related injuries in 2019 vs. 2020.

There was no statistical difference (p=0.697) in management (non-operative or operative) between the two study periods. However, the trend of increase in operative management during the pandemic was noted (Figure 4). 

## Discussion

The COVID-19 pandemic lockdown in the UK changed the pattern of orthopedic workload referrals. This study reports a 35.6% decrease in total adult orthopedic referrals during the study period. This is corroborated by multiple studies that have reported a reduction in the total number of referrals to orthopedics from 43% to 58% [9-11]. The reduction in the total number of referrals is likely to be multifactorial and may be secondary to people adhering to the national lockdown measures during the study period, limiting travel outside, and a reduction or cessation of organized sporting activities. Within the hospital, departmental changes were initiated including having senior decision-makers in emergency departments, which may also have helped to reduce the total number of patients referred during this time. 

In contrast, the number of cycling-related injuries referred to the proportion of total orthopedic referrals was seen to increase with statistical significance in the present study. There are a number of possible causative factors for this pattern. It is likely that with people adhering to lockdown and avoiding public transport, cycling uptake increased. This was incentivized by the government and was seen as a way to reduce individual COVID-19 risk. As a result, it follows that there would be an increase in cycling injuries with more people using this as both a form of transport and exercise. In addition, more inexperienced cyclists started to use this as a mode of transport. MacDonald et al. studied patients requiring surgery in five orthopedic units in Scotland during the pandemic, noting an increase in patients with cycling-related injuries requiring surgery during lockdown [12]. Our study noted a trend toward more operative management during the pandemic; however, it was not statistically significant. This is reassuring as it demonstrates that the pandemic did not change the orthopedic management of these injuries. 

The average age of injured cyclists was 34.8 years in 2019 and 31.5 in 2020. Previous studies have demonstrated that the majority of cycling collisions occur in men aged 45-50 years [7,13]. In this study, there was an increase in the number of female cyclists injured indicating that more women were taking up cycling during the lockdown. This change in the demographics of cycling-related injuries is corroborated by further evidence from the fitness-tracking app Strava [14]. In the UK, activities recorded by women on Strava rose by 108% for females aged between 18 and 29 years during 2020. The reason for the increase in female participation in cycling may be due to changes in perception of transport safety, cycle safety, and improved access to bicycles. The increase in injuries in younger cyclists could be explained by a desire to avoid public transport while commuting to work or other essential travel. This age group may well have also felt more confident leaving the house during lockdown than the older cohorts. 

In this study, the number of cyclist vs. vehicle collisions did not change during the lockdown; this we felt was an unusual observation as the department for transport reported that car and bus traffic decreased by 24.7% and 32.0%, respectively, in 2020 [15]. They also noted decreases in van and lorry traffic of 9.1% and 5.7%, respectively. An interesting observation is that bicycle traffic increased by 45.7%. We speculate that the unchanged amount of cyclist vs. vehicle collisions may be due to there being more cyclists on the road, with fewer vehicles, along with the fact that more novice cyclists took up cycling during lockdown.

A statistical difference was observed in the relative increase in upper limb trauma during the pandemic. The reason for this increase is less obviously answered by the pandemic. We propose that lower-energy injuries are more likely to result in a fall off the bicycle onto an outstretched arm, whereas higher-energy injuries may result in lower limb/polytrauma. It may be that novice cyclists have a higher incidence of low-energy cycling collisions resulting in upper limb trauma. 

Following the pandemic, public behaviors regarding travel are likely to be different. There has been a significant uptake in cycling, which has been recognized and supported by the government. The health and economic benefits of cycling are well known. Commuting by bicycle has advantages over other modes of transport, both for the commuter and for society [16]. If we continue to observe this increasing trend of people cycling then this will impact the amount of cycling-related injuries presenting to hospitals. Pathways to facilitate the timely intervention and management of these injuries will need to be implemented. If patterns remain as this study reports, the majority of patients requiring surgery will have upper limb injuries and will be able to go home prior to their surgery taking place. Day case surgery provision will be beneficial to manage this workload. In addition to the changes seen in the hospital setting, there will also be a need for continued support from the government to try and make cycling as safe as possible for the population, with additional cycle lanes, car-free zones, cycling proficiency workshops as well as highlighting to other road users the need to be aware of more cyclists being on the road. 

Limitations

This study represents the admissions of one level 1 MTC in London during the COVID-19 pandemic. At times, there was a change in pre-hospital triage depending on hospital capacity. Although we assume referrals are accurate, we cannot account for those referrals that may have been redirected to other hospitals. In addition, this is a retrospective study, so the data collected is limited. Other contributing variables such as the cyclists' level of experience could not be assessed. 

## Conclusions

This study demonstrates a significant difference in the number of patients presenting to the orthopedic department with cycling-related injuries since the COVID-19 pandemic began. It evidences a significant increase in upper limb trauma following cycling injuries during the pandemic. The study provides evidence suggesting that cycling-related injuries continued to increase during lockdown. An adaptation of orthopedic services was needed to cover the increase in upper limb trauma requiring surgery. Further retrospective work is required to see if this pattern is observed at other centers in the UK. In addition, it would be interesting to see if similar patterns are seen in cities that have become "cycling-focused" post-pandemic. 
